# General Practitioner Supervisor assessment and teaching of Registrars consulting with Aboriginal patients – is cultural competence adequately considered?

**DOI:** 10.1186/1472-6920-14-167

**Published:** 2014-08-13

**Authors:** Penelope Abbott, Jennifer Reath, Elaine Gordon, Darshana Dave, Chris Harnden, Wendy Hu, Emma Kozianski, Cris Carriage

**Affiliations:** 1 School of Medicine, University of Western Sydney, Locked Bag 1797, Penrith NSW 2751, Australia; 2 Northern Territory General Practice and Training, Penrith, Australia

## Abstract

**Background:**

General Practitioner (GP) Supervisors have a key yet poorly defined role in promoting the cultural competence of GP Registrars who provide healthcare to Aboriginal and Torres Strait Islander people during their training placements. Given the markedly poorer health of Indigenous Australians, it is important that GP training and supervision of Registrars includes assessment and teaching which address the well documented barriers to accessing health care.

**Methods:**

A simulated consultation between a GP Registrar and an Aboriginal patient, which illustrated inadequacies in communication and cultural awareness, was viewed by GP Supervisors and Medical Educators during two workshops in 2012. Participants documented teaching points arising from the consultation which they would prioritise in supervision provided to the Registrar. Content analysis was performed to determine the type and detail of the planned feedback. Field notes from workshop discussions and participant evaluations were used to gain insight into participant confidence in cross cultural supervision.

**Results:**

Sixty four of 75 GPs who attended the workshops participated in the research. Although all documented plans for detailed teaching on the Registrar’s generic communication and consultation skills, only 72% referred to culture or to the patient’s Aboriginality. Few GPs (8%) documented a plan to advise on national health initiatives supporting access for Aboriginal and Torres Strait Islander people. A lack of Supervisor confidence in providing guidance on cross cultural consulting with Aboriginal patients was identified.

**Conclusions:**

The role of GP Supervisors in promoting the cultural competence of GP Registrars consulting with Aboriginal and Torres Strait Islander patients could be strengthened. A sole focus on generic communication and consultation skills may lead to inadequate consideration of the health disparities faced by Indigenous peoples and of the need to ensure Registrars utilise health supports designed to decrease the disadvantage faced by vulnerable populations.

## Background

Aboriginal and Torres Strait Islander Australians have markedly worse health and lower life expectancy than other Australians [1]. To close the gap on this health disadvantage, health care must be high quality, accessible, well-coordinated, targeted to individual needs and culturally competent [2]. Cultural competence is a lifelong professional process in which health practitioners consider culture as a factor impacting on health care, incorporate self-reflection into every patient interaction and seek to decrease health disparities by actively improving care for people from disadvantaged and diverse populations [3-5].

Cultural competency training will always be a simplification of complex issues [6,7]. Culture can vary between different communities of the same ethnic background and changes over time. It cannot be easily disentangled from social factors which impact on individuals and communities, including the socioeconomic disadvantage faced by Aboriginal and Torres Strait Islander communities today as a result of their history of colonial oppression [8,9].

Despite the inherent complexity of facilitating learning in cultural competence, it remains important for equitable health care. Cultural competence can increase over time when respectful attitudes are combined with increasing cultural awareness and self-reflection, including consideration of the need to recognise racism as a barrier to care and to address health disparities [6,10].

In Australia, vocational general practice (GP) training is delivered through Regional Training Providers (RTPs), and includes hospital and community components. GP Registrar is the term used to describe a postgraduate doctor enrolled in the GP training scheme. GP Registrars undertake at least two years of community training placements, comprising terms of 6 to 12 months in several general practices, usually under the supervision of experienced GP Supervisors within the same practice [11]. The Supervisors provide tutorials and opportunistic teaching to Registrars, and oversee the quality of their care, sharing responsibility with Registrars for the care of the Registrars’ patients [12]. Additional mentorship and workshop-style teaching is provided by RTP-based Medical Educators, who are GPs with educational experience [13]. Supervisors and Medical Educators are also involved in Registrar assessment, such as in external clinical teaching visits and in college clinical examinations.

GP training curricula require Registrars to understand Aboriginal and Torres Strait Islander history and culture and how this impacts on health and relationships with healthcare providers [14,15]. A small number of Registrars undertake training placements at Indigenous Health Training Posts (IHTPs) [13]. These posts are usually based in multidisciplinary Aboriginal community controlled health services, where Aboriginal health workers, nurses, doctors and allied health workers provide culturally appropriate primary health care [16]. The supervision of Registrars in these posts has a strong focus on ensuring the cultural safety of patients, and GP Supervisor teaching is supported by Aboriginal and/or Torres Strait Cultural Educators (CEs) and Mentors (CMs) [17].

Outside IHTPs, the number of Aboriginal or Torres Strait Islander patients seen by Registrars is variable and may be affected by suboptimal identification of Aboriginal or Torres Strait Islander status [18]. Although all Registrars are required to attend some formal cultural education, usually in the form of a cultural awareness workshop, there are no current mandated systems to provide cultural supervision of Registrars who see Aboriginal and Torres Strait Islander patients in non-IHTP placements. In areas of Australia where the practice proportion of Aboriginal and Torres Strait Islander people is small, the task of assessing and supporting Registrar cultural learning needs usually falls by default to their GP Supervisors or Medical Educators, who may have limited knowledge and experience themselves. The purpose of this study was to examine the confidence and skills of non-Indigenous GP Supervisors in providing feedback to a GP Registrar consulting with an Aboriginal patient using a video of a simulated consultation within a cultural training workshop.

## Methods

The University Of Western Sydney Human Research Ethics Committee (H9254) and the Aboriginal Health and Medical Research Council Ethics Committee (811/11) approved this study.

### Background to the development of the simulated consultation video resource

We used literature evidence and interviews with Aboriginal CEs and CMs to create a video resource of a simulated consultation illustrating cross-cultural learning points. The video-resource was designed as a trigger for a cultural training session for GPs. In a previous study [19], CE and CM advice on what GPs needed to work effectively with Aboriginal patients, including common errors which GPs were seen to make, was incorporated into the resource. This resource was viewed and endorsed for training use by the CEs and CMs and by the two Aboriginal community controlled health services in our training region. In the role play, an Aboriginal patient, accompanied by an older relative, presents for management of a sore shoulder, at which time the GP also attempts to provide opportunistic care for the patient’s diabetes. The GP has good clinical knowledge and wants to assist his patient, but lacks the skills to do this effectively, subsequently demonstrating multiple instances of poor communication and lack of cultural awareness. The purposely highlighted cultural errors included use of inappropriate terminology around Aboriginality, reference to skin colour as a determinant of Aboriginality, failure to understand the role of the Elder and lack of acknowledgement of the significance of cultural references and protocols which the patient discusses in the consultation. In another important feature of the video, the GP Registrar does not utilise national health programs and supports which aim to decrease health disparities in Aboriginal and Torres Strait Islander people, despite evident financial and logistic barriers to care. In particular the Registrar does not discuss with the patient the ‘Closing the Gap’ program. This national government initiative substantially decreases the cost of medication and provides access to Aboriginal case workers and to transport, thus supporting the healthcare needs of Aboriginal people with or at risk of chronic disease [20].

### Video-feedback workshop and data collection

We led workshops with two RTPs at non-mandatory GP Supervisor training days in 2012. We collected informed consent from GP Supervisors and Medical Educators through verbal and written advice that the aim of the research was to investigate their confidence and skills in assessing and giving feedback to a non-Indigenous GP Registrar consulting with an Aboriginal patient, as shown in a simulated consultation.

A Cultural Educator and GP member of the research team facilitated the workshops. After viewing the simulated consultation, participants filled out a ‘Consultation assessment form’ (Table  1) in which they noted the strengths and weaknesses of the consultation and the teaching points for subsequent feedback to the Registrar. These were collected for later analysis. The workshop then proceeded into an interactive large group session in which the Cultural Educator answered questions and gave specific advice on the observed cultural errors and omissions. Members of the research team took field notes, including descriptions of the discussion content and participant quotes. At the conclusion of the workshop, participants provided comment on the workshop and research process on a written Evaluation form. All data collected were anonymous.

**Table 1:** Consultation assessment form

*Please make specific comments in relation to the following areas*	What did the GP registrar do well?	What could be improved?
Communication and rapport		
History and management		
Other skills relevant to the consultation		
*Additional questions:*		
• What process would you follow in debriefing this consultation?		
• What questions would you ask of your registrar when providing feedback?		
• What would you use from this consultation as teaching points to discuss in a feedback session with your GP registrar?		
• What other educational resources may be useful for this registrar?		

### Data analysis

We used a content analysis approach for the qualitative data from the Consultation assessment forms. In this methodology, all instances of the occurrence of interest are coded, allowing analysis of the frequency of the occurrence as well as analysis of concepts emerging from the data (16). We coded the data as (a) relating to general communication and consultation skills, as would apply to all clients regardless of culture or ethnicity, and (b) feedback which specifically referred to culture and Aboriginal health. Five members of the research team contributed to the identification of the key concepts arising from the data. Two members of the research team coded the data, comparing and agreeing on categorisation. We coded data from all fields of the Consultation assessment forms to capture the overall content of the GP Supervisor’s planned feedback.

Examples of feedback which we coded within the general communication and consultation skills category include verbal and non-verbal communication; listening skills; a respectful, non-judgemental approach; and reference to managing the patient in their context without giving any details connecting this to the Aboriginal context or mentioning culture. The category relating to explicit discussion of culture and/or Aboriginal health included any use of the word culture; any use of the term Aboriginal; any identification of cultural errors; and discussion of health supports for Aboriginal people. Also included in this category was some content we agreed implicitly referred to principles of cultural competence or to the patient’s culture, even if not explicitly stated. We further examined these over-arching categories as to whether feedback was detailed or general. We coded feedback as general when GP Supervisors simply noted a need for improved communication skills or improved cultural sensitivity but did not comment further, and as detailed if at least one specific area of planned feedback was noted. Content relating to the clinical assessment and management of the patient, such as comments on the medical aspects of diabetes care, was not coded for analysis.

We collated and summarised the workshop field notes and Evaluation forms to assess the usefulness and acceptability of the workshop and to further examine the GPs’ confidence in supervising Registrars in their consultations with Aboriginal patients. This study adheres to the RATS guidelines for reporting qualitative studies.

## Results

In total, 71 GP Supervisors and 4 Medical Educators attended the two workshops. We did not collect data on the characteristics of the GP participants, who were all involved in GP training in urban and large rural settings. Two of the 75 workshop participants identified themselves to the workshop facilitators as working in an Aboriginal community controlled health service which was an IHTP. Sixty-eight participants consented to take part in the study and of these 64 submitted completed Consultation assessment forms, thus comprising the study group. Results of the content analysis are presented in Table  2.

**Table 2:** Identified learning and teaching needs to be discussed in feedback session

**Category**	**Frequency**	**Interview data (RTP A & B)**
**General teaching points**		
Communication and consultation skills	100% identified this as an area requiring improvement, and all gave at least one specific detail to be addressed in feedback session	‘Less didactic, paternalistic approach’ (A3)
		‘Respectfulness – meet the patient on the same level as a participant on the same journey’ (A4)
		‘Locus of control remained with doctor – no likelihood of improved management or self care’ (A6)
		‘Criticising patient too much’ (B25)
		‘Listen- patient agenda not GP agenda’ (A15)
		‘Not patient- centred’ (A16)
		‘Bad basic consultation skills for any patient’ (A10)
		‘Doctor needs to check patient understanding before going on to other issues’ (B36)
		‘No open ended questions to allow her to discuss her concerns’ (B41)
Cultural awareness and understanding	72% (48/64) identified a need for the registrar to better consider the patient’s culture or Aboriginality within the consultation	‘Understanding of Aboriginal culture & awareness’ (A4)
		‘Lacks exploration of patient’s many social and cultural factors and needs’ (B37)
		‘Cultural respect and safety’ (B45)
		‘Once you identified the patient as Aboriginal what other cultural issues did you need to address?’ (A9)
	66% (42/64) gave at least one specific detail related to the Aboriginal context or Aboriginal health to be addressed in registrar feedback *(see below)*	
**Specific teaching points relevant to culture or Aboriginal health**		
Cultural issues impacting on the consultation	42% (27/64) - Cross cultural communication/consultation	‘Explore ways in which he may alter his management style to be more culturally appropriate’ (A3)
	28% (18/64) - Identification of and terminology around Aboriginality	‘Made judgement about Aunt’s Aboriginality without asking. “Part Aboriginal” doesn’t exist’ (A5)
		‘Understanding of the meaning of Aboriginality’ (A10)
	27% (17/64) - The significance of Elders and family, and how to manage in a consultation	‘Didn’t acknowledge or thank the Aunty’s presence, support and contribution’ (B29)
	17% (11/64) - Ensure lifestyle advice is culturally appropriate	‘Do you think an Aboriginal lady, short of money…plays tennis?’ (A1)
		‘Assumption of appropriateness and usefulness of handouts’ (B9)
	14% (9/64)- Sorry business (bereavement)	‘Acknowledgment of the ‘Sorry’ event that had affected her life’ (B33)
		‘Acknowledge demands of Sorry business’ (A6)
Health	9% (6/64) - Health issues of particular importance in Aboriginal people	‘Awareness Aboriginal higher risk of having cardiac disease’ (B14)
Supports to assist patient to manage heath needs	14% (9/64) - Access Aboriginal healthcare providers and services	‘Try to involve Aboriginal health worker and support.’ (B15)
		‘Referral to Aboriginal services/dietitian who have better understanding of Aboriginal culture.’ (B17)
		‘Referred to allied health - didn’t check re transport issues’ (A5)
	8% (5/64)- Identified the relevance of government funded initiatives to support health care costs and support needs (Closing the Gap program)	‘Failure to appreciate money – costs of meds. CTG could be used.’ (B41)
**Supporting ongoing learning in Aboriginal health**		
Response to question ‘What educational resources may be helpful?’	20% (13/64) - cultural awareness learning resources (including specific reference to workshops, written and visual materials)	‘Aboriginal awareness training’ (A4)
		A day at Aboriginal health centre. Reading material. Video.’ (A3)
	16% (10/64) - accessing cultural advice and mentorship from Aboriginal people	‘Discussion with Aboriginal Health Worker. Sitting in on consultation with mentor.’ (A5)
	8% (5/64) - recommended Registrar needed improved knowledge of Aboriginal history	‘History of Aboriginal self-determination. Many GPs weren’t born or educated in Australia – are unaware of the stolen generation, referendum Aboriginal vote’ (B16)

All participants gave detail on communication as a teaching priority and the large majority documented their planned feedback on the Registrar’s inadequate communication skills in comprehensive detail. Cultural or Aboriginal health-related factors which impacted on the quality and effectiveness of the consultation were recorded by 72% (48/64) of participants, and 66% (42/64) identified at least one issue specific to cross-cultural consultations or to the patient’s context as an Aboriginal person which they would raise with the Registrar.

Specific teaching points relating to cultural and Aboriginal health understanding are detailed in Table  2. Most participants did not document planned teaching on cultural errors in the consultation or on facilitation of health care for the patient given her Aboriginality, despite the patient specifically stating she could not afford her medications and referring to other barriers to accessing health care. Involving Aboriginal healthcare providers or services was documented by 14% (9/64) of participants. Only 8% (5/64) of participants mentioned the national government initiative, the ‘Closing the Gap’ program, as a point to discuss in subsequent teaching. Few participants (11% or 7/64) identified Aboriginal health resources or resource people to whom they would direct the Registrar, despite this being a specific question in the Consultation assessment form.

Almost all participants stated they would promote a respectful approach to the patient. There was no specific documentation of planned feedback encouraging self-reflection on potential cultural bias or discussion of racism, except for 2 GPs who referred to ‘humility’ and the concept of a ‘dominant culture’ , which was likely to preface feedback to the Registrar on this theme.

The field notes from the large group discussions were congruent with the Evaluation forms and the content analysis of the Consultation assessment forms, evidencing both the well-developed confidence and skills of GP participants in teaching communication and consultation skills, and their lesser confidence and skills in assessing and supporting the cultural competence of Registrars consulting with Aboriginal patients.

There was some polarisation of participant views around the content of the simulated consultation and the teaching plans they had documented on the Consultation assessment forms. In the view of some GPs, the communication errors in the consultation were too blatant and took away from the cultural focus of the workshop, preventing it from being a useful learning exercise. Some expressed that this was why they did not mention culture or Aboriginal health in their planned feedback to the Registrar. Other participants held an opposing view, commenting that the video-tool had been a thought-provoking trigger to discussion, prompting them to consider whether they had placed adequate emphasis on cultural issues in the consultation and leading to self-reflection on their role in assessing and promoting cultural competence in their Registrars. These divergent views were clear in participant comments:

“*There is a question for me - debriefing the communication was easy, but I am not sure how to work with this debriefing about culture”*

“*I would find this teaching tool very limited as there was so much to be improved in terms of overall consulting skills that the focus on Aboriginal cultural issues would be lost”*

“*I have a feeling we are all really focused on the consultation skills”*

## Discussion

Most GP Supervisors and Medical Educators who took part in this study confidently identified generic communication and consultation learning needs in the simulated consultation and the need for a respectful approach. Most, but not all, referred to learning needs around cultural issues, however this generally lacked detail. This was in contrast to sophisticated detail on the teaching needed to promote communication and consultation skills. Strikingly, few documented a need to assist the patient through national programs and community supports for Aboriginal and Torres Strait Islander people as a teaching priority.

It is well known that structural barriers to equitable health care exist. Governmental, organisational and societal barriers to provision of accessible and culturally competent care can prevent health practitioners from providing effective care even if they strive to be culturally competent as individuals [6]. However, the converse is also true – health practitioners need to assist their patients to access available health initiatives which are designed to tackle health disparities in order for such programs to be effective. In the context of our study, teaching about doctor-patient communication appeared to overshadow consideration of cultural awareness, barriers to accessing health care and the availability of the Closing the Gap program. This may reflect a lack of explicit consideration of the impact of the patient’s cultural background on their access to health care.

Training which promotes cultural competence can be undervalued throughout medical training, influenced by a hidden curriculum in which biomedical knowledge is the key to success [21]. Teaching and supervising communication and consultation skills as well as medical competence are core and explicit roles of the GP Supervisor and Medical Educator. However, they are not necessarily trained to reflect on the cultural competence of their Registrars or practiced at debriefing consultations which demonstrate learning needs in managing Aboriginal patients. Some GP Supervisors may feel they are not in a position to provide advice on Aboriginal cultural issues if they themselves are not an Aboriginal person with accepted skills in cultural education or mentorship. However, cultural competence is a key aim of GP training, and Aboriginal and Torres Strait Islander educators are not usually available to provide opportunistic advice or supervision within GP training practices outside the IHTP network at the present time. Thus, many GP Supervisors will be directly faced with addressing this key training issue when Registrars are in need of guidance and during assessment of Registrar performance.

Many participants documented feedback which would promote patient-centred care for the patient, without further highlighting culture or the patient’s Aboriginality. Patient-centred care is a well-recognised principle of general practice, in which the patient perspective is respected and the GP and patient find common ground to achieve more effective healthcare and better health outcomes [22]. Culturally competent care is aligned to patient-centred care in terms of a respectful approach and a shared commitment to healthcare which is effective and acceptable to individuals in their context. However, it goes beyond this to explicit recognition of the power differential that exists between the dominant culture and other peoples and an active commitment to equity of care [5,6]. Patient-centred care and cultural competence are both important to improve cross-cultural communication, patient satisfaction and health outcomes [23].

The professional uncertainty and disempowerment clinicians can feel when dealing with cultural diversity has been previously identified [24]. Family physicians may be reluctant to mention or consider culture within consultations for risk of stereotyping patients [3]. Cultural competence is a complex area which encompasses more than acknowledgement of ethnicity, and having some cultural awareness does not ensure health care providers have adequate understanding [25]. Training which aims to promote cultural awareness and cultural competence can instead risk promoting detrimental conceptualisations of those from non-dominant cultures as ‘different’ , thus further perpetuating bias [26]. However fear of stereotyping can lead to a failure to acknowledge the impact of a person’s sociocultural background on their health (including facing racism in life and in healthcare) and a lack of self-reflection as to the healthcare provider’s own cultural assumptions [3,4]. The approach where healthcare providers and services aim to provide the same care to all clients regardless of culture and ethnicity has been termed ‘cultural blindness’ and, although often well intentioned, favours the clients most assimilated into the dominant culture and can lead to healthcare providers overlooking opportunities to reduce health disparities [4,5]. This may have been an attitude influencing our research findings. The Supervisor focus on patient-centred care and on avoiding stereotyping rather than on a cultural competence approach to the consultation may explain why so little teaching was planned on cultural awareness and overcoming barriers to care, despite supportive systems being available.

Teaching is likely to be enhanced by specifically encouraging reflection on culture, both as it applies within the consultation and also in a societal context. The impact of culture on health and GP management when the patient is from a non-dominant culture, including the widening of power differentials within the doctor-patient relationship and barriers to care related to racism and unequal access to health services, deserves explicit consideration. Perceived preparedness to provide cross-cultural care was found in a US study of junior doctors to be related to the amount of training received and access to role models they considered good at providing cross-cultural care [27]. Supervisors with higher cultural competence have been found to deliver increased supervision in cultural competence to their supervisees [28].

Despite the complexities of what is necessarily lifelong learning for cultural competence, advice on how to avoid communication misunderstandings and provide health services for Aboriginal patients can support GPs to work more effectively with Aboriginal people [19,29]. Experience working with Aboriginal patients is likely to increase the cultural competence of Registrars, but requires supervision that identifies cultural errors, encourages active consideration of the role of culture within consultations with Aboriginal people and guides the Registrar to learn more about Aboriginal health, including how to access supports to overcome health disparities. Though best supported by Aboriginal Cultural Educators and Mentors, cultural competence should be considered at every teaching and assessment opportunity in GP training, and falls within the domain of teaching provided by GP Supervisors when Registrars consult with Aboriginal patients in their training placements.

### Limitations

Our study has several limitations. The simulated consultation was specifically designed to illustrate cultural errors and omissions that could be made by non-Indigenous GPs when providing health care for Aboriginal patients, and was authorised as appropriate by the Aboriginal Cultural Mentors and organisations involved in its development. However, poor communication skills may have overwhelmed the lack of cultural competence demonstrated in the consultation.

Collected data on planned teaching comprised only that which GP Supervisors and Medical Educators documented they would intend to deliver when guiding the Registrar and may not have reflected the actual teaching they would give the Registrar, nor the level of detail which would eventuate. Exploration of communication issues and promotion of a patient-centred approach may have brought out other issues relating to culture or Aboriginal health for discussion. However, the coding categories we used in the content analysis were intentionally broad and required simply a mention of culture or Aboriginality to be seen as signifying the Supervisor would provide teaching around cultural competence and Aboriginal health, thus attempting to allow for these limitations. Furthermore, the cross cultural nature of the workshop and the purpose of the study were not hidden from participants. They were aware it was an activity to examine their skills and confidence in assessing Registrar consultations with Aboriginal patients, so the finding that a significant proportion of participants did not document a plan which referred to the patient’s Aboriginality or culture may suggest a lack of consideration of relevant cultural issues.

In seeking to investigate teaching priorities for Registrars consulting with Aboriginal patients, we chose to examine the explicit consideration of culture and Aboriginal health, an evident simplification of a complex area. Appropriate communication and medical management are also an integral part of an effective cross cultural medical encounter.

## Conclusion

GP Supervisors are skilled and confident at assessing general consultation and communication factors which impact on consultations between Registrars and Aboriginal patients. However they appear less likely to provide feedback that specifically considers the impact of the patient’s culture on their healthcare and to advocate for use of readily accessible national support programs designed to overcome health disparities for Aboriginal people. The role of GP Supervisors in promoting the cultural competence of GP Registrars consulting with Aboriginal patients outside specialised training posts could be strengthened. A sole focus on generic communication and consultation skills may lead to inadequate consideration of the health disparities faced by Aboriginal people, and of the need to ensure that GP Registrars utilise heath supports designed to decrease this disadvantage.

## Competing interests

The authors declare we have no competing interests.

## Authors’ contributions

PA, CH, JR and EG conceived of the study, and the project was designed by PA, JR, EG, WH and CH. Aboriginal cultural advisors to the project were EG and CC. The video resource and workshop was developed and implemented by PA, DD, EG and JR. Data collection and analysis was done by PA, EK, WH, JR and EG. PA wrote the first draft of the paper. All authors reviewed the paper and approved the final draft.

## Pre-publication history

The pre-publication history for this paper can be accessed here:

http://www.biomedcentral.com/1472-6920/14/167/prepub
